# The distinctive material cycle associated with seabirds and land crabs on a pristine oceanic island: a case study of Minamiiwoto, Ogasawara Islands, subtropical Japan

**DOI:** 10.1007/s00442-025-05725-0

**Published:** 2025-05-22

**Authors:** Nozomu Sato, Rumiko Nakashita, Tetsuro Sasaki, Hidetoshi Kato, Haruki Karube, Hideaki Mori, Kazuto Kawakami

**Affiliations:** 1 Graduate School of Urban Environmental Sciences, Tokyo Metropolitan University, 1-1 Minami-Osawa, Hachioji, Tokyo 192-0397 Japan; 2 Forestry and Forest Products Research Institute (FFPRI), 1 Matsunosato, Tsukuba, Ibaraki 305-8687 Japan; 3 Institute of Boninlogy, Nishi-Machi, Chichijima, Ogasawara, Tokyo 100-2101 Japan; 4 Faculty of Science, Tokyo Metropolitan University, 1-1 Minami-Osawa, Hachioji, Tokyo 192-0397 Japan; 5 Kanagawa Prefectural Museum of Natural History, 499 Iryuda, Odawara, Kanagawa 250-0031 Japan; 6 Japan Wildlife Research Center, 3-3-7 Kotobashi, Sumida, Tokyo 130-8606 Japan; 7 Hokkaido Research Center, Forestry and Forest Products Research Institute, 7 Hitsujigaoka, Toyohira, Sapporo, Hokkaido 062-8516 Japan

**Keywords:** Alien species, Food web, Restoration, Stable isotope, Volcano Islands

## Abstract

Seabirds are responsible for transporting marine material to oceanic islands, and attempts are being made to restore their function on many islands where they have become extinct. However, little is known about the original island ecosystems prior to disturbance. Minamiiwoto, located in the Ogasawara Islands, is an uninhabited oceanic island that remains uninvaded by alien animals, and its pristine ecosystem and material cycle should serve as a reference for the restoration of disturbed island ecosystems. We analyzed the food web structure of several of the Ogasawara Islands with different disturbance intensities using stable isotopes (δ^13^C, δ^15^N) and compared the characteristics of the material cycle. We found that seabirds and land crabs are distributed across the entire island of Minamiiwoto, with high δ^15^N values derived from marine resources and a gradient in the δ^15^N of land crabs reflecting differences in seabird species with elevation. In contrast, on islands where forest-nesting seabirds have been extinct for more than 50 years, the nutrient supply to the island interior has been lost, and the δ^15^N of most organisms was significantly lower. Isotopic food niches among predators were clearly partitioned by species (max. 14% overlap) on Minamiiwoto, while on the disturbed islands they tended to be highly similar (max. 53% overlap). Our results confirmed that Minamiiwoto still maintains a pristine ecosystem characterized by material transport by seabirds and decomposition by land crabs. The recovery of these biological functions should be the guide for conservation and restoration of oceanic islands subjected to anthropogenic disturbance.

**Supplementary Information:**

The online version contains supplementary material available at 10.1007/s00442-025-05725-0.

## Introduction

Oceanic islands have never been connected to continental lands, allowing the evolution of unique biota isolated from mainland communities (Wallace 1881; Carlquist 1974). The transport of material from the marine environment to land by seabirds is a crucial factor for the establishment of island ecosystems. Seabirds transport nutrients through food leftovers, droppings, and carcasses, favoring vegetation and animal colonization (Polis and Hurd 1996; Polis et al. 1997; Mulder and Keall 2001; Fukami et al. 2006; Otero et al. 2018). These transported nutrients contribute to the formation of phosphorus- and nitrogen-rich soils on islands (Fukami et al. 2006; Otero et al. 2018). Furthermore, the runoff nutrients supplied from islands to the ocean represent the basis for the high productivity of coral reef ecosystems (Graham et al. 2018). Seabirds are ecosystem engineers that alter the physical environment of island litter and soils influencing the diversity of other communities (Orwin et al. 2016).

Around the world, many oceanic islands have now lost their seabird populations and ecosystem functions provided by them due to human settlement and associated alien species (such as rats and feral cats), and consequently the bird-mediated marine-derived nutrient supply has also been lost (Pitman et al. 2005; Loss et al. 2013; Harper and Bunbury 2015; Graham et al. 2018). Rats not only prey directly on island organisms but also significantly alter the island ecosystem functions through indirect impacts on above- and below-ground communities (Fukami et al. 2006). Small seabirds weighing less than 300 g are particularly vulnerable to the negative impacts of rats (Jones et al. 2008). For this reason, measures to control alien species are being aggressively implemented and, in many islands, seabird breeding grounds and terrestrial decapod crustacean communities have been successfully recovered after the eradication of feral cats and rats (Buxton et al. 2014; Jones et al. 2016; Nigro et al. 2017; Brooke et al. 2018). However, after the eradication of rodents, the populations of seabirds that recover are dominated by highly migratory cosmopolitan species, and it is difficult for less migratory species that nest in forests to re-establish themselves; therefore, the seabird fauna and its ecosystem functions are different from those before the invasion of the alien species (Kawakami and Horikoshi 2022). It is difficult to restore native biota just by eradicating alien species, but efforts should be made to restore ecosystem functions to a level that is as close as possible to the original conditions. Previous studies of insular material cycles have examined only environments subjected to anthropogenic disturbance and where eradication measures had been implemented, but there is little knowledge of a pristine oceanic island ecosystems. To our knowledge, there have been no observations of island material cycles in a completely undisturbed environment in the last few hundred years (even the Chagos archipelago had undergone some anthropogenic disturbance decades before the survey: MacNeil et al. 2015; Graham et al. 2018).

Therefore, elucidating the food web structure of a pristine oceanic island can assist in evaluating the recovery of ecosystem functions after the eradication of alien species. It is presumed that the nutrients transported by seabirds accumulate in the soil for a certain amount of time and that there is a time lag between the extinction of seabirds and the loss of their function. However, it remains unknown for how long these nutrients are retained in the environment after seabird extinction. The Ogasawara Islands, a marine archipelago comprising more than 30 islands located 1000 km from mainland Japan in the Pacific Ocean, can serve as a model for these issues. These islands include Minamiiwoto, which has no human settlements or rat invasions, and a total of 21 seabird species have been recorded as breeding there (Bryan 1903; Momiyama 1930; Kawakami and Horikoshi 2022); however, many that were present on each island in the nineteenth century became extinct due to overhunting and alien species (e.g. black rat and feral goat) (Kawakami and Horikoshi 2022). The timing of extinction varies among islands, providing useful information for comparisons the impact of seabird loss.

Here, we investigated how the history of the invasion of alien species and the loss of seabird populations has changed the characteristics of material cycles and the structure of food webs on oceanic islands. The species composition of breeding seabirds on Minamiiwoto vary depending on altitude (Kawakami 2019), so we also evaluated the changes in material circulation that this causes. Firstly, we hypothesize that the length of time that seabirds were lost from the islands would be reflected in the nitrogen cycle of the island’s organisms, with a decrease in marine-derived nutrients (nitrogen) across the islands. Secondly, it is predicted that the niches of food resources available to terrestrial animals have changed significantly and deviated from the original food web because many seabirds and invertebrates have become extinct on the inhabited islands where many alien species have invaded.

## Materials and methods

### Study sites

The Ogasawara Islands (Fig. S1 in Online Resources) have a subtropical climate, and the only native mammal species present is the Bonin flying fox (*Pteropus pselaphon*), while native snakes or amphibians are absent (Kawakami 2008). Ogasawara Islands are divided into two main archipelagos: the Bonin Islands and the Volcano Islands.

Chichijima (27° 4′ 40″ N, 142° 13′ 0″ E) and Hahajima (26° 39′ 50″ N, 142° 9′ 30″ E), which are part of the Bonin Islands, have been inhabited and their lands have been cultivated since around 1830 (for approximately 190 years). Before human disturbance, these islands would have been breeding grounds for many seabirds, but now, seabirds are almost extinct due to the impact of alien black rats and feral goats (Chiba et al. 2007; Horikoshi et al. 2009). Therefore, it is likely that seabird breeding sites on these islands disappeared quickly after human settlement.

Kitaiwoto (25° 26′ 0″ N, 141° 16′ 55″ E), in the Volcano Islands, was inhabited from about 1900 until 1944 during World War II (WWII) and has remained uninhabited for more than 70 years (Kawakami et al. 2021). Rats have invaded the island, and only a small number of boobies (*Sula sula* and* Sula leucogaster*) and red-tailed tropicbird (*Phaethon rubricauda*) now breed on the coast, while several shearwaters (*Puffinus*) and storm-petrels (*Oceanodroma*) which breed in the forest, became locally extinct with the last sightings dating back to the 1930s (Chiba et al. 2007; Kawakami et al. 2021).

Minamiiwoto (24° 14′ 2″ N, 141° 27′ 49″ E) is located in the southernmost part of the Volcano Islands and is one of the Natural Monuments of Japan, where aircraft landings are prohibited. The entire island is mountainous with steep terrain and has never been settled by humans (Suzuki et al. 2018; Kawakami 2019). The only mammal is the flying fox, and terrestrial decapods dominate as the second largest animal group in terms of body size after birds (Sasaki et al. 2018). Several surveys of the island’s biota showed that the environment remained pristine, with numerous endemic species and few alien plants. The pristine Volcano Islands would have been widely covered with forests, with petrels being the predominant bird species (Momiyama 1930; Rich et al 1986; Ohashi et al. 2024). Further detailed information on each island is available in Online Resources.

### Sample collection and stable isotope analysis

Carbon and nitrogen stable isotope ratio analysis was used to visualize the food web structure of each island and to estimate the effects of nutrients from seabirds and disturbance by invasive species. As an example, the δ^15^N values of organisms living on rat-free islands are several times higher than the δ^15^N values of organisms living on rat-invaded islands (Hobson et al. 1999; Caut et al. 2008; Fukami et al. 2006; Graham et al. 2018).

Specimens of representative seabirds, land birds, rats, lizards, terrestrial crustaceans, insects and plants were collected on Chichijima, Hahajima, Kitaiwoto, and Minamiiwoto, which are the main island ecosystems of the Ogasawara archipelago, to conduct stable isotope analyses. Seabird specimens were collected only on Minamiiwoto, while rat specimens were collected only on the other islands. Many of the migratory birds recorded in the Ogasawara Islands are irregular migrants with small populations (Hasuo 1980), and as they do not play a major role in the island’s food web. For this reason, they were not included in our sampling. Sampling was mainly carried out in June to account for the effects of seasonal changes in stable isotope ratios. Specifically, on Chichijima and Hahajima, almost all plants and animals were collected in June 2017 or June 2019 at elevations ranging from 0 to 440 m. Specimens of endemic lizards (*Cryptoblepharus nigropunctatus*) were collected on Hahajima from October to November 2017. Feather samples were obtained from seabird specimens collected on Chichijima and Hahajima in June from 1999 to 2013, considering that the molting period for these birds is mainly from May to September. On Kitaiwoto and Minamiiwoto, samples were collected in June 2008, 2009, and 2019 at elevations ranging from 0 to 616 m and in June 2007, 2016, and 2017 at elevations ranging from 0 to 900 m, respectively.

All samples were completely dried at 60 °C for 24 h. The plant samples, which consisted of one leaf taken from each plant, were dried and then crushed. The crustacean, rat, and lizard specimens had their muscles removed by dissection and were degreased using the chloroform + methyl alcohol method. Bird feathers were cleaned and degreased. The crushed whole bodies of arthropod specimens were used for analysis. Samples were enclosed in a tin cup and combusted in a FlashEA1112 elemental analyzer (Thermo Fisher Scientific, Bremen, Germany) interfaced to a Delta V isotope ratio mass spectrometer (Thermo Fisher Scientific). To compare isotope ratios in food webs and species on each island, the δ^13^C or δ^15^N content in each sample was calculated according to the following equation:

$$\delta^{13} C \, or \, \delta^{15} N \, \left( \permille \right) \, = \, \left( {R_{{{\text{sample}}}} /R_{{{\text{standard}}}} - 1} \right) \, \times \, 1000 \, \left( {R \, =^{13} C/^{12} C,^{15} N/^{14} N} \right)$$

where R_sample_ is the abundance ratio of the sample, R_standard_ is the international standard, Vienna Pee Dee Belemnite, and atmospheric nitrogen. The analytical errors for the isotope analysis were within 0.1‰ for δ^13^C and 0.2‰ for δ^15^N.

### Characteristics of the food web structure and δ^15^N content in each Island

To investigate the food web variations in the pristine and disturbed environments, the overall communities on each island were compared based on the δ^13^C and δ^15^N values. The samples were categorized into the following nine types by food source, feeding habit, and taxon: seabirds (n = 15), land birds (60), lizards (85), rats (22), crustaceans (83), herbivorous insects (43), detritivorous insects (29), C4 plants (53), and C3 plants (278) (Tables S1, S2 in Online Resources). The following numbers of animal and plant species were used for isotope analysis, respectively: 12 and 6 on Minamiiwoto, 9 and 5 on Kitaiwoto, 13 and 7 on Chichijima and Hahajima. For animals, 2–29 individuals per species were analyzed for each island, and for plants, 2–40 individuals were analyzed.

The δ^15^N values of species common among the islands were used to estimate the effects of seabird-derived nutrients and alien rodents; comparisons were made for six animal species (*C. nigropunctatus, Coenobita purpureus, G. grayi, Horornis diphone, Hypsipetes amaurotis,* and *Zosterops japonicus*) and five plant species (*Callicarpa subpubescens, Ficus nishimurae, Machilus kobu, Miscanthus* species, and *Pandanus boninensi*)*.* Since Chichijima and Hahajima have very similar disturbance histories and biota, the data obtained from them were analyzed together via inter-island comparisons.

Because the values of the Minamiiwoto samples were highly influenced by elevation, those collected above 500 m were excluded so as to be able to make inter-island comparisons with the Bonin Islands samples, which were collected at lower elevations. Furthermore, to investigate the effect of elevation on δ^15^N in Minamiiwoto, three species distributed along the entire elevational gradient were compared, i.e., *C. nigropunctatus, G. grayi,* and* Miscanthus sinensis.*

### Comparisons of isotopic niche spaces

Niche overlap was evaluated by analyzing isotopic niche space to assess the impact of non-native organisms on native organisms as well as the shifts in the food habits of multiple consumers associated with environmental changes (Nigro et al. 2017; Domingo et al. 2020). We used Stable Isotope Bayesian Ellipses in R (SIBER, Jackson et al. 2011) to analyze the width of feeding niches between species or communities based on stable isotope ratios. The total area of a convex hull (TA) is represented by the polygonal area using the outer shell of all plots. TA is strongly influenced by outlying observations and the number of samples (Layman et al. 2007). Therefore, the small sample size collected standard ellipse area (SEAc), which is based on 40% of the data close to the centroid, is generally used (Batschelet 1981). Here, the overlapping of SEAc represented the isotopic niche width of each vertebrate and invertebrate predator (limited to a sample of at least five individuals), and the percentage of niche sharing was calculated for all combinations using the SIAR package in R 4.3.0 (R developmental core team, 2023). Samples at all elevations were used in the analysis.

### Statistical analysis

The post-hoc comparison with Bonferroni correction or Welch t-test was used to compare the isotopic ratios (δ^13^C, δ^15^N) between same species on two or three islands (α = 0.05). Changes in nitrogen stable isotope ratios with elevation were analyzed using a generalized linear model (Gaussian or negative binomial distribution) with δ^15^N as the response variable and elevation as the explanatory variable. All analyses were performed in R 4.3.0 (R Developmental Core Team, 2023).

## Results

### Analysis of Island communities

#### Chichijima and Hahajima

**Fig. 1:**
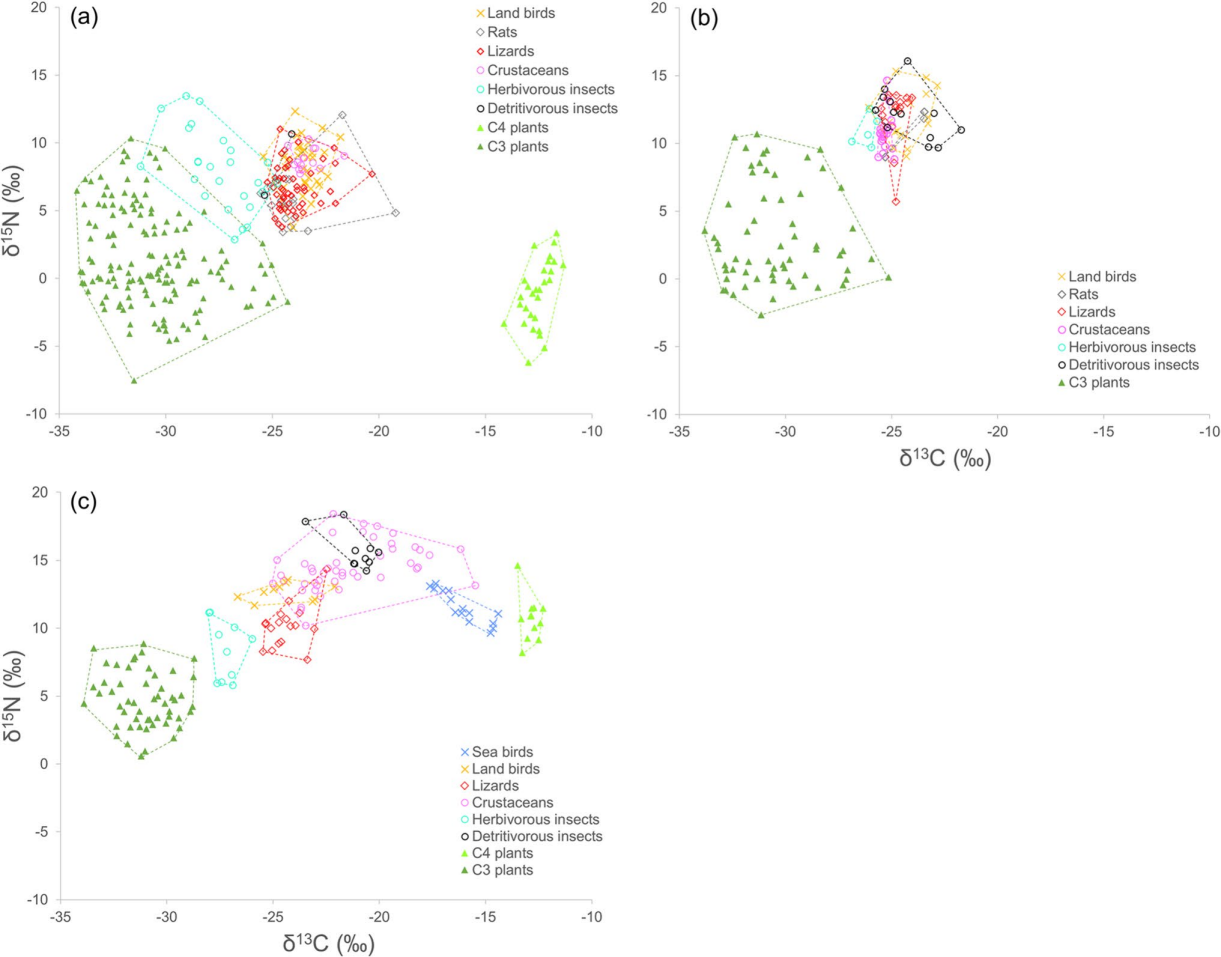
Food web structures based on stable isotope analysis (δ^13^C, δ^15^N) in three communities of the Ogasawara Islands. **a** Chichijima and Hahajima, **b** Kitaiwoto, **c** Minamiiwoto. The dashed lines indicate the total area of the convex hull for each group of organisms

The following averages and ranges of stable isotope ratios were obtained for the animal and plant species sampled in Chichijima and Hahajima: C3 plant samples (δ^15^N mean: 1.5‰, δ^15^N range: – 7.5 to 10.3‰, δ^13^C mean: – 30.6‰, δ^13^C range: – 34.2 to – 24.3‰, *n* = 151), animal samples (7.5‰, 2.9–13.5‰, – 24.4‰, – 31.2 to – 19.2‰, *n* = 145) (Fig. 1a; Tables S1, S2). The δ^15^N values obtained in these two islands were on average 3.2‰ and 5.4‰ lower than those in Minamiiwoto, respectively, and 1.6‰ and 4.1‰ lower than those in Kitaiwoto, respectively. The δ^15^N values of land birds, lizards, and rats were comparable with those of native herbivorous insects, while the δ^13^C values were different (δ^15^N mean ± SD: 7.7 ± 1.2‰, δ^13^C mean ± SD: – 27.3 ± 1.8‰, *n* = 21).

#### Kitaiwoto

The following averages and ranges of stable isotope ratios were obtained for the animal and plant species sampled in Kitaiwoto: C3 plant samples (δ^15^N mean: 3.1‰, δ^15^N range: – 2.7 to 10.7‰, δ^13^C mean: – 30.4‰, δ^13^C range: – 33.8 to – 25.1‰, *n* = 62), animal samples (11.6‰, 5.7–16.1‰, – 24.8‰, – 26.9 to – 21.7‰, *n* = 76), with the values for each animal group tending to overlap within a very narrow range (Fig. 1b). The δ^15^N values were on average 1.6‰ and 1.3‰ lower than those detected in Minamiiwoto, respectively.

#### Minamiiwoto

The following averages and ranges of stable isotope ratios were obtained for the animal and plant species sampled in Minamiiwoto: C3 plant samples (δ^15^N mean: 4.7‰, δ^15^N range: 0.6 to 8.9‰, δ^13^C mean: − 31.0‰, δ^13^C range: – 33.9 to – 28.7‰, *n* = 53), animal samples (12.9‰, 5.8–18.4‰, – 21.7‰, – 28.0 to – 14.4‰, *n* = 97) (Fig. 1c). High isotope contents were detected in crustaceans (δ^15^N mean ± SD: 15.0 ± 1.5‰, δ^13^C mean ± SD: – 20.9 ± 2.3‰, *n* = 36) and detritivorous insects (15.7 ± 1.3‰, – 21.1 ± 0.9‰, *n* = 10), with the δ^13^C values of these groups being higher than those of other terrestrial animal groups but close to those of seabirds (11.6 ± 1.2‰, – 16.9 ± 1.1‰, *n* = 15). The δ^13^C values of C4 plants ( – 12.9 ± 0.4‰, *n* = 12) were significantly different from those of C3 plants; however, C4 plant contribution to the food web was low because *M. sinensis* is almost exclusively confined to summit areas on Minamiiwoto.

### Inter-island comparison of nitrogen isotope ratios by species

**Fig. 2:**
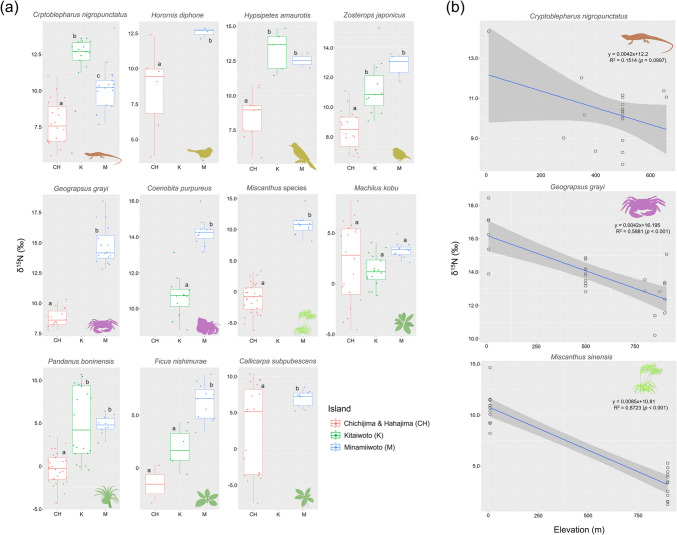
Boxplot of δ^15^N values for species shared among islands and variation of δ^15^N with elevation in Minamiiwoto. **a** Intraspecies comparisons between islands. Different letters indicate significant differences detected via post-hoc comparison with Bonferroni correction or Welch t-test (*p* < 0.05). **b** δ^15^N elevation gradient at Minamiiwoto. The area shaded in dark gray indicates the 95% confidence interval

Five native animal species, *Z. japonicus* (post-hoc comparison with Bonferroni correction, *p* < 0.001), *H. diphone* (*p* < 0.001), *C. nigropunctatus* (*p* < 0.001), *H. amaurotics* (Welch *t*-test, *p* < 0.05), and *G. grayi* (*p* < 0.05) showed significantly higher δ^15^N contents in Minamiiwoto than in Chichijima and Hahajima (Fig. 2a; Table 1). *H. amaurotis* and *C*. *nigropunctatus* showed the highest δ^15^N value in Kitaiwoto (post-hoc comparison with Bonferroni correction, *p* < 0.05). The δ^15^N of *C. purpureus* in Minamiiwoto was higher than that in Kitaiwoto (*p* < 0.001). There was no significant difference between Kitaiwoto and Minamiiwoto in *H. amaurotis* and *Z. japonicus* (*p* = 1.00, *p* > 0.10 respectively).

**Table 1:** Stable nitrogen isotope ratio (^15^N/^14^N) of animals and plants in three island groups

Species	Minamiiwoto		Kitaiwoto		Chichi & Haha		M vs K		M vs CH		K vs CH	
	*n*	δ^15^N (‰)	*n*	δ^15^N (‰)	*n*	δ^15^N (‰)	*t*	*p*	*t*	*p*	*t*	*p*
Animals												
*Horornis diphone*	3	12.6 ± 0.3		–	6	8.5 ± 2.8	-	-	– 3.172	**0.023**	–	–
*Hypsipetes amaurotis*	2	12.5 ± 0.5	5	13.2 ± 1.3	10	8.5 ± 1.5	0.553	1.000	– 3.343	**0.014**	– 5.574	** < 0.001**
*Zosterops japonicus*	5	12.8 ± 0.7	11	11.2 ± 1.7	18	8.5 ± 1.3	– 2.007	0.160	– 5.927	** < 0.001**	– 5.001	** < 0.001**
*Cryptoblepharus nigropunctatus*	19	10.2 ± 1.6	17	12.1 ± 2.0	20	7.8 ± 1.5	3.273	**0.006**	– 4.205	** < 0.001**	– 7.609	** < 0.001**
*Coenobita purpureus*	11	14.3 ± 0.7	12	10.7 ± 1.1		-	– 9.067	** < 0.001**	–	–	–	–
*Geograpsus grayi*	26	14.8 ± 1.6		-	15	8.8 ± 0.7	–	–	– 13.218	** < 0.001**	–	–
Plants												
*Miscanthus* species	12	10.7 ± 1.6		–	29	–1.1 ± 2.4	–	–	– 15.514	** < 0.001**	–	–
*Machilus kobu*	12	3.3 ± 0.7	16	1.2 ± 1.5	20	2.1 ± 4.0	– 1.953	0.171	– 1.199	0.711	0.918	1.000
*Callicarpa subpubescens*	10	6.9 ± 1.1		–	19	2.5 ± 6.2	–	–	20.318	**0.007**	–	–
*Pandanus boninensi*	10	4.8 ± 1.0	20	5.0 ± 4.1	29	– 0.3 ± 1.8	0.195	1.000	– 4.976	** < 0.001**	– 6.537	** < 0.001**
*Ficus nishimurae*	11	6.2 ± 1.8	6	1.9 ± 1.7	2	– 1.6 ± 1.8	– 4.584	** < 0.001**	– 5.409	** < 0.001**	– 2.243	0.118

Mean ± Standard deviation. Post-hoc comparison with Bonferroni method used for three groups, or Welch *t* test used for two groups (α = 0.05). Bold indicates the significant differences

In plants, δ^15^N contents tended to be higher in less disturbed islands (Kitaiwoto and Minamiiwoto) than in inhabited islands (Chichijima and Hahajima) (Fig. 2a; Table 1). Four of the five native plant species, i.e., *M. boninensis* (Welch *t*-test, *p* < 0.05), *C. subpubescens* (*p* < 0.05), *P. boninensis* (post-hoc comparison with Bonferroni correction, *p* < 0.001), and *F. nishimurae* (*p* < 0.001), showed higher δ^15^N values in Minamiiwoto than in Chichijima and Hahajima. Conversely, some samples from *M. kobu* showed higher δ^15^N values on Chichijima and Hahajima than on the other islands (*p* > 0.10). *F. nishimurae* from Kitaiwoto tended to show higher values than those from Chichijima and Hahajima but was not significant (*p* > 0.10). For *P. boninensis*, the values for Minamiiwoto and Kitaiwoto were almost the same (*p* = 1.00).

### The effect of elevation on δ^15^N

The variations in nitrogen stable isotope ratios with elevation on Minamiiwoto were significant for two of the five species sampled in this study. The δ^15^N values of *G. grayi* (generalized linear model: estimate ± SD = – 0.0042 ± 0.0007, z value = – 5.854, *p* < 0.001) and *M. sinensis* ( – 0.0014 ± 0.0002, z value = – 6.595, *p* < 0.001) were high in coastal areas and tended to be lower at higher elevations (Fig. 2b). The δ^15^N values of *C. nigropunctatus* did not change with elevation ( – 0.0042 ± 0.0024, z value = – 1.742, *p* = 0.0997), but only individuals collected in coastal areas showed higher values.

### Isotopic niche overlaps between high-order consumers

#### Chichijima and Hahajima

**Fig. 3:**
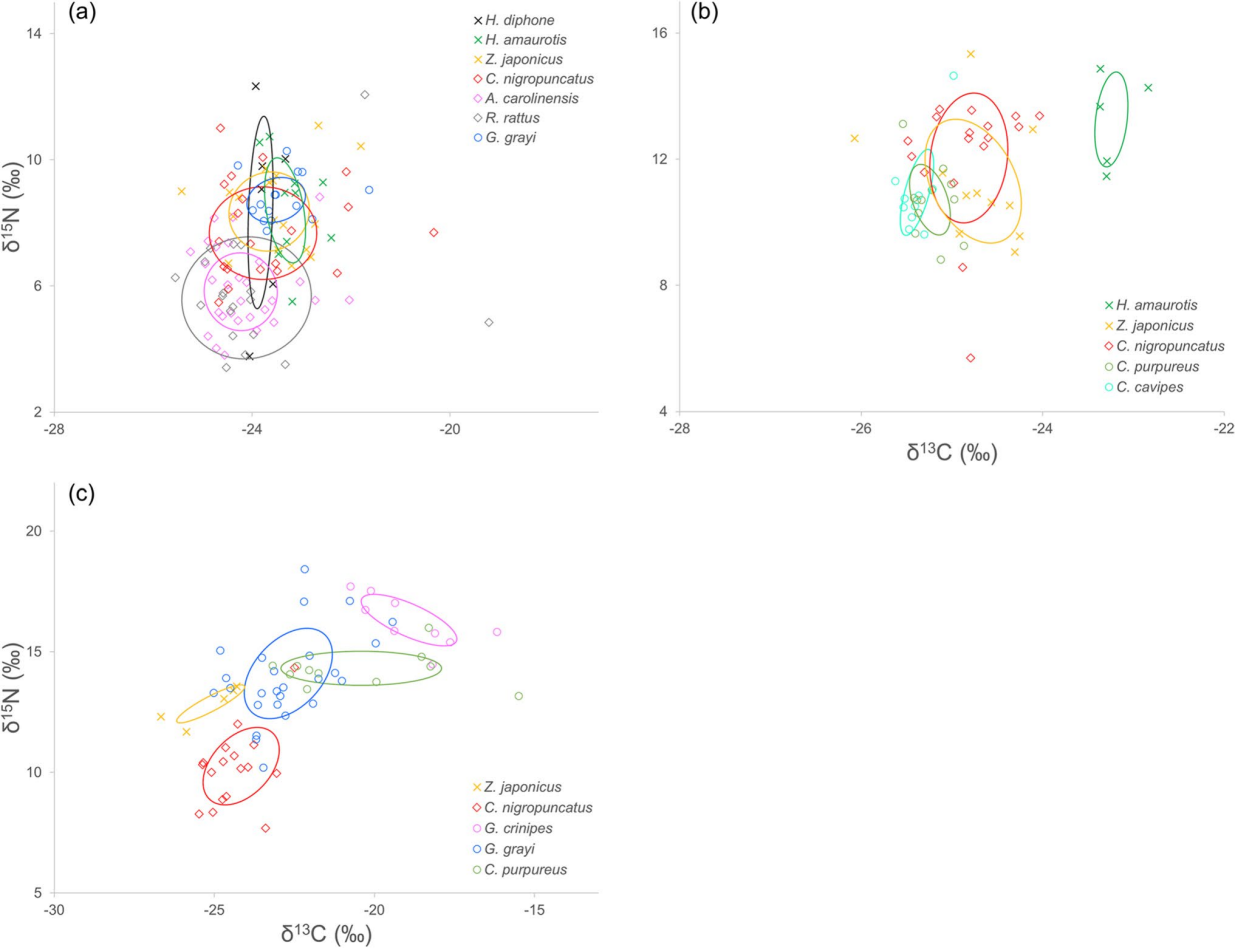
Isotopic niche (SEAc) overlaps among high-order consumers. **a** Chichijima and Hahajima, **b** Kitaiwoto, **c** Minamiiwoto. The circle for each species indicates the standard ellipse area (SEAc) for small sample sizes

The highest SEAc overlap between consumers showed high resource similarity not only among native species but also with non-native species (Fig. 3a). The three land bird species examined showed an overlap of 7.9% to 43.5% with each other and an even greater overlap with *C. nigropuncatus* and *G. grayi* (22.7–46.2% and 17.0–43.5%, respectively) (Table S3). Alternatively, these land birds exhibited a low overlap with the alien green anoles and black rats (0–13.3% and 3.3–10.3%, respectively). The native lizard *C. nigropuncatus* shared 10.8%, 18.9%, and 21.0% of its niche with green anoles, black rats, and *G. grayi*, respectively. Green anoles exhibited the highest overlap (35.8%) with black rats, while no overlap at all was observed between *G. grayi* and the black rats or anoles (both 0%).

#### Kitaiwoto

When examining the five native animal species, the overlap between *C. nigropuncatus* and the two land hermit crabs (*C. cavipes* and *C. purpureus*) was low (5.7 and 1.3%, respectively). There was a 26.8% overlap between these two species of hermit crabs, indicating similar resources (Fig. 3b; Table S4). *Z. japonicus* also exhibited a low overlap with *C. purpureus* and *C. cavipes* (7.0% and 3.0%), and its diet was very similar to that of lizards (52.6% overlap). In contrast, *H. amaurotis* showed stable isotope ratios that were different from those of all the other consumers, including *Z. japonicus*, with a 0% SEAc overlap in all cases.

#### Minamiiwoto

For each of the five native animal species examined, the isotopic niche spaces of high-order consumers (land birds, lizards, and terrestrial crustaceans) were separated, with an SEAc ellipse overlap of 0% in most cases (Fig. 3c; Table S5). Only the land crabs *G. grayi* and *C. purpureus*, which feed on seabird and marine fish carcasses, showed a 14.3% overlap. The species at the highest trophic level (δ^15^N: 16.3 ± 1.0‰) in this food web was *G. crinipes*.

## Discussion

### Material cycles on the Ogasawara Islands

In Kitaiwoto, where burrow-nesting seabirds have disappeared for more than 50 years, δ^15^N were on average about 1.3‰ and 1.6‰ lower in animals and plants, respectively, than those observed in Minamiiwoto. In Chichijima and Hahajima, where seabirds have been extinct for more than 150 years, a fourfold and twofold change in animals and plants, respectively, was observed compared with the values detected in Kitaiwoto, as shown by the significant decrease in δ^15^N concentrations throughout the food web. This indicates that Minamiiwoto in the Ogasawara Islands has never been settled by humans or disturbed by alien species, and that the material cycle of nutrients supplied by the marine is maintained. Furthermore, it supports our hypothesis that the length of the time during which seabirds were lost is reflected in the nitrogen cycle of the organisms, with a decrease in marine-derived nitrogen across the island. The largest difference in this parameter was observed in land crabs, reaching a maximum of 10.4‰ between Minamiiwoto and Chichijima. The land crabs of Minamiiwoto are “climbing crabs” that travel from the coast to mountain summit and back for spawning (Sasaki et al. 2018). They function as top predators and decomposers that feed on seabird carcasses or eggs, and their high trophic level (denoted by the δ^15^N values) supported this observation.

The isotope ratios of marine fish in the Pacific Ocean near Japan are 6 to 11‰ for δ^15^N: and – 17 to – 18‰ for δ^13^C (Ohshimo et al. 2023), and such values were reflected in the feathers of seabirds on Minamiiwoto. Land crabs may enhance material cycles on this island because terrestrial decapods generally contribute to the formation of island vegetation by feeding on plants, decomposing carcasses and litter, dispersing seeds, and supplying marine nutrients (Lee 1985; O’Dowd and Lake 1989; Green et al. 1999; Pitman et al. 2005; Paulay and Starmer 2011). Furthermore, because land crabs do not prey on seabirds (unlike rats), without interfering with the function of seabirds to transport marine nutrients, and support the dispersal of nutrients. On islands where rats live, land crabs only live in coastal areas (Sasaki et al. 2021), so these functions have been lost in most islands in the Ogasawara.

### Variation of stable isotope ratios with elevation

Stable isotope ratios can vary with elevation under the influence of topography and other complex factors, such as climate and biota. Due to changes in temperature and precipitation, the δ^15^N content in soil and plant leaves tends to decrease with elevation (Houlton et al. 2007; Liu and Wang 2010). Such decrease tends to be stronger in C4 plants than in C3 plants (Liu and Wang 2010), so it is likely that the variations in δ^15^N on Minamiiwoto are driven by climatic conditions. However, the δ^15^N elevational gradient in *G. grayi*, a carrion-feeding species, may reflect differences in the distribution of nutrient-supplying seabirds. Because different seabird species use different resources (fish prey, feeding grounds), the characteristics of the nutrients supplied to the islands are likely to vary depending on seabird species composition, size, and other factors. For example, in the Chagos Islands, large differences in nitrogen inputs have been detected between islands with different seabird species and abundances (Graham et al. 2018). On Minamiiwoto, the distribution of seabird species tends to vary with elevation and forest cover. For example, grassland- and ground-nesting seabirds (large size) such as the red-tailed tropicbird and brown booby nest along the coast and in lowlands below 300 m of elevation, while burrow-nesting seabirds (small size) such as the Bonin petrel, Matsudaira’s storm petrel, and Tristram’s storm petrel nest at altitudes above 400 m (Kawakami 2019). Larger seabirds are generally found at higher trophic levels, while smaller seabirds tend to feed more frequently on food at a lower trophic level (Hobson et al. 1994), and this difference in diet is possibly responsible for the differences in δ^15^N with elevation. This segregation based on differences in seabird body weight, flight and competition abilities, and nesting characteristics determines the supply of nutrients to the entire island and also the differences in nutrient characteristics.

### Ecosystem changes associated with disturbance levels

On Chichijima and Hahajima, where seabird breeding grounds have been lost for more than 150 years, isotopic niches among consumers extensively overlapped. On the Bonin Islands, the significantly lower isotopic diversity of high-order consumers compared to native plants along with the different δ^13^C values obtained for predators and native herbivore insects suggested that the impact of alien species has reduced the food resources available to consumers. On these two islands, many native invertebrate populations have declined due to alien species (e.g., green anoles), and new interactions have formed among them (Sugiura 2016). Previous analyses of the stomach contents of green anoles have shown a predominance of hemipteran insects that parasitize non-native plants, which suggested that these lizards became dependent on non-native insects because of the decline of many native insects (Toda et al. 2010; Takahashi et al. 2014). In this study, the isotopic niches of green anoles and black rats extensively overlapped but differed in that anoles are arboreal and insectivores, while rats are omnivores, feeding primarily on plant matter and ground invertebrates (90% plant matter on Chichijima, Yabe 1979). In addition, although competition- and predation-related conflicts with native lizards have been reported, anoles tend to feed on larger prey (Toda et al. 2010), and no significant resource overlap was detected between the isotopic niches of the two species. As for land birds, the Japanese white-eye (*Z. japonicus*) and brown-eared bulbul (*H. amaurotis*) are omnivores that feed mainly on insects and fruits; however, on inhabited islands, they more frequently consume the fruits of alien plants, consequently dispersing their seeds (Kiyosu 1978; Kawakami et al. 2008). Hence, the isotopic niches of these land birds were similar to each other and also overlapped with the niche of lizards. Although stable isotopic niches do not necessarily reflect a true dietary niche, the similarities observed among these consumer species with different dietary habits may be due to the limited available food resources.

On Kitaiwoto, where forest-nesting seabird populations have been lost for more than half a century, seabird colonies and crabs persist only in coastal areas, and marine nutrients are not supplied to soils at high elevations and inland. Nutrients have been lost as a result of topsoil runoff on islands invaded by feral goats, but forests have been preserved on Kitaiwoto (Ohashi et al. 2024), and it is presumed that nutrients in its forest soils are still retained. This may cause a time lag in the degradation of the material cycle. The ranges of the δ^15^N value of native plants showed intermediate properties between Minamiiwoto and Bonin Islands. The δ^15^N values of *C. nigropunctatus*, *H. amaurotis*, and *Pandanus boninensis* samples collected on the coast of Kitaiwoto were higher than Minamiiwoto, suggesting the influence of the nutrient supply by ground-nesting seabirds in the coastal area. Although the number of samples examined was not sufficient, the small isotopic range and high similarity between animal groups may indicate that, like on Chichijima and Hahajima, the food resources available to native land birds and lizards of Kitaiwoto are limited due to the impact of rodents.

In contrast to these two islands that have been disturbed for a long time, Minamiiwoto exhibited little niche overlap due to the variety of available food resources and the resource partitioning of each consumer. This rodent-free environment was characterized especially by a high density of *G. grayi* and the presence of the predatory crab *G. crinipes*. In addition to hermit crabs, terrestrial decapods (e.g., Gecarcinidae) are also vulnerable to alien rats (Pitman et al. 2005; Nigro et al. 2017; Sasaki et al. 2021). These land crab populations have been shown to significantly recover after rat eradication (Bellingham et al. 2010; Paulay and Starmer 2011; Nigro et al. 2017), and intra-guild predation makes them the species with the highest trophic level in island ecosystems (Nigro et al. 2017). Nigro et al. (2017) reported that feeding niches and habitats shared between crustaceans expanded, and a greater overlap between species was detected after rat eradication in Palmyra Atoll (average elevation, only 2 m). However, on Minamiiwoto, no niche overlap between the two land crab species examined was detected. This was possibly due to differences in ecological and geographic characteristics, such as the fact that *G. crinipes* tends to prey on other crustaceans in coastal areas, whereas *G. grayi* is more widespread on mountain tops of up to 916 m of altitude, where it has access to a wider range of food resources (e.g., seabird eggs and carrion) and a larger activity area (Sasaki et al. 2018). Because increasing crab populations alter the ecological cycles and litter decomposition patterns on islands (O’Dowd and Lake 1989; Green et al. 1999; Lindquist et al. 2009), it will be worth to examine the variations in food resources used by *G. grayi* from the coast to mountain tops as well as the changes in ecosystem functions with elevation.

## Conclusions

Our results suggested that climbing land crabs have an important role in the decomposition and dispersal of these marine nutrients. This study documented the occurrence of long-term ecosystem changes, especially after the disappearance of burrow-nesting seabirds, which are the most important species responsible for the nutrient supply to island interiors. Thus, the material cycle in the primary ecosystems of subtropical oceanic islands is characterized by an abundance of ecosystem engineers with specific functions and by distinct resource partitioning among native consumers. Although restoring a destroyed oceanic island ecosystem to its original state is a difficult task, recovery of the populations and functions of forest-dwelling seabirds and land crabs should be one guiding principle in oceanic island ecosystem conservation and restoration projects.

## Supplementary Information

Below is the link to the electronic supplementary material.

Supplementary file1 (DOCX 144 KB)

## Data Availability

Raw data are available in Figshare (https://doi.org/10.6084/m9.figshare.25513855).
