# Supplementary material for: The distinctive material cycle associated with seabirds and land crabs on a pristine oceanic island: a case study of Minamiiwoto, Ogasawara Islands, subtropical Japan
Source: Oecologia. 2025 May 22;207(6):88. doi: 10.1007/s00442-025-05725-0 (PMC12098187; doi:10.1007/s00442-025-05725-0)
Supplement: Supplementary file 1 — Supplementary file1 (DOCX 144 KB) [file 442_2025_5725_MOESM1_ESM.docx]

ELECTRONIC SUPPLEMENTAL MATERIAL

The distinctive material cycle associated with seabirds and land crabs on a pristine oceanic island: a case study of Minamiiwoto, Ogasawara Islands, subtropical Japan

Authors: Nozomu Sato*, Rumiko Nakashita, Tetsuro Sasaki, Hidetoshi Kato, Haruki Karube, Hideaki Mori, Kazuto Kawakami

*Study sites*


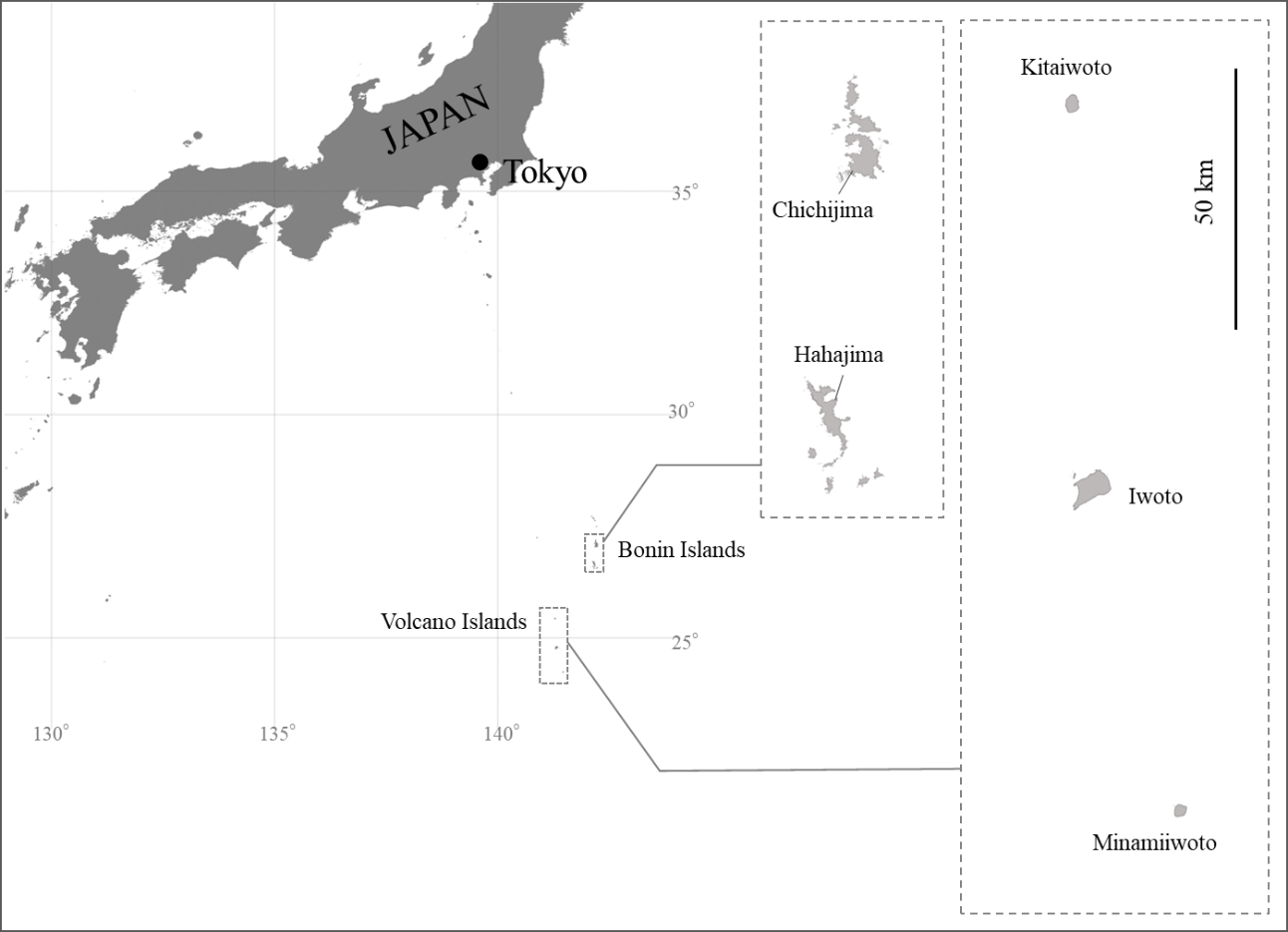


Figure S1 Map of the Ogasawara Islands, Japan.

The base map was provided by the Geospatial Information Authority of Japan (https://www.gsi.go.jp/top.html).

Bonin Islands

Chichijima (total area: 23.45km^2^, maximum elevation: 326 m; Fig. S1) and Hahajima (19.88 km^2^, 463 m) are both inhabited islands located approximately 36 km apart. Chichijima was occupied in the 1830s, and arable land increased rapidly from the 1880s to 1900 (Katahira 1981). Hahajima was cultivated from 1876 to early 1900, and most of the territory, except for some mountainous areas, was turned into fields and residential land (Katahira 1982). Chichijima has lost much of its surface soil due to feral goats, cultivation, and wars that destroyed the vegetation on most of the island (Hiradate et al. 2015). In addition, many native invertebrates have been wiped out following the introduction of alien species such as green anole (*Anolis carolinensis*), cane toad (*Bufo marinus*), New Guinea flatworm (*Platydemus manokwari*), and land nemertean (*Geonemertes pelaensis*) (Kishimoto 2009; Karube 2010; Abe et al. 2010; Chiba and Cowie 2016; Sugiura 2016; Shinobe et al. 2017). Five species of introduced mammals (feral goat, feral cat, black rat, Norway rat, and house mouse) are present on Chichijima, and over 300 species of alien vascular plants have been recorded (Suzuki and Suzuki 2009; Kawakami 2019a). Numerous seabird fossils (e.g., *Pterodroma hypoleuca*, *Calonectris leucomelas*) have been excavated on the Bonin Islands, where seabird communities thrived before human disturbance (Ono 1984; Kawakami and Horikoshi 2022). Records of seabird breeding since the 19th century are limited to small-scale activity detected on the islands’ margins (Seebohm 1890; Momiyama 1930; Chiba et al. 2007). Currently, only two seabird species breed on Hahajima, in a small area on one of its peninsulas (Horikoshi et al.2009). Terrestrial crustaceans (Decapoda) are mainly restricted to coastal areas and low elevations, and purple-backed shore crab (*Geograpsus grayi*) is rare (Sasaki et al. 2021). Thus, it is estimated that more than 150 years have passed since the loss of nutrient supply by seabirds to the interior of these two islands.

Volcano Islands

Kitaiwoto, Iwoto, and Minamiiwoto form the Volcano Islands. Kitaiwoto (area 5.57 km^2^; maximum elevation, 792 m) is an uninhabited island with few flat areas and mostly steep and heavily eroded terrain. Fields have been cultivated on this island since around 1900, and people lived here until 1944, when they were evacuated (Kawakami et al. 2021). While the Kitaiwoto shares many plant species with the Bonin Islands, it has been colonized by more than 30 alien plant species (Kato and Goto 2021) and by alien mammals, i.e., black rats and Norway rats (Kawakami et al. 2021). Eight seabird species were breeding on the island until WWII, but now only three large species (boobies and tropicbirds, with a body weight above 800 g) breed on the coast, and burrow-nesting seabirds (e.g., petrels, with a body weight below 400 g) have disappeared due to the impact of rodents (Chiba et al. 2007, Kawakami et al. 2021). Although eight land crab species (seven hermit crabs and *G. grayi*) have been recorded, their distribution is limited to coastal areas (at elevations below190 m, Sasaki et al. 2021). Furthermore, although petrels were breeding until 1939 (Yamashina Institute for Ornithology specimen database <https://decochan.net>, accessed on Nov. 14, 2023), their presence has not been recorded for more than 80 years, and they are likely to have become locally extinct in the mid-20th century. Therefore, it was estimated that about half a century has passed since the island lost the nutritional supply derived from these seabirds.

Minamiiwoto (area 3.54 km^2^, maximum elevation, 916 m) is entirely mountainous and, like Kitaiwoto, steep and heavily eroded. Cloud forests exist above 500 m (Shumiya et al. 2018). The Bonin flying fox is the only native mammal, and no rodents are present on the island. A total of nine seabird species are found in the area, the largest number in all of the Ogasawara Islands (Chiba et al. 2007; Kawakami et al. 2018). Seabird nesting is dense in forests and coastal vegetation, with species weighing less than 300 g nesting along the entire elevational gradient, from low elevations to mountain summits (Kawakami et al. 2018; Kawakami 2019b). Six species of land crabs (three hermit crabs and three *Geograpsus* crabs) are found on the island, with *G. grayi* being distributed along the entire elevational gradient (Sasaki et al. 2018).

**References**

Abe T, Makino SI, Okochi I (2010) Why have endemic pollinators declined on the Ogasawara Islands? In: Kawakami K and Okochi I (eds.) Restoring the Oceanic Island Ecosystem: 75-83. Springer, Tokyo.

Chiba H, Kawakami K, Suzuki H, Horikoshi K (2007) The distribution of seabirds in the Bonin Islands, southern Japan. J Yamashina Inst Ornith 39(1): 1-17.

Chiba S, Cowie RH (2016) Evolution and extinction of land snails on oceanic islands. Annu Rev Ecol Evol Syst 47: 123-141.

Hiradate S, Morita S, Hata K et al (2015) Effects of soil erosion and seabird activities on chemical properties of surface soils on an oceanic island in Ogasawara Islands, Japan. Catena 133: 495-502.

Horikoshi K, Suzuki H, Sasaki T, Chiba Y (2009) The impact assessment of invasive alien mammals on sea bird colonies. Chikyu Kankyo 14(1): 103-105. (in Japanese)

Karube H (2010) Endemic insects in the Ogasawara Islands: Negative impacts of alien species and a potential mitigation strategy. In: Kawakami K and Okochi I (eds.) Restoring the Oceanic Island Ecosystem: 133-137. Springer, Tokyo.

Katahira H (1981) Characteristics of the Land Use Situation on Chichijima: Focusing on Prewar Land Use. In: Report on the Survey of the Natural Environment in the Ogasawara Islands: 155-162. Tokyo Metropolitan University Press, Tokyo. (in Japanese)

Katahira H (1982) Cultivation and distribution of farm land in Hahajima: Focusing on Prewar Land Use. In: Report on the Survey of the Natural Environment in the Ogasawara Islands: 141-144. Tokyo Metropolitan University Press, Tokyo. (in Japanese)

Kato H, Goto H (2021) Vascular plant flora of Kita-iwoto Island. Ogasawara Res 47: 73-92. (in Japanese with English summary)

Kawakami K, Suzuki H, Horikoshi K, Kawaguchi D (2008) Avifauna of Minami-Iwo-To Island, the Volcano Islands in 2017. Ogasawara Res 33: 91–123. (in Japanese with English summary)

Kawakami K, Suzuki H, Horikoshi K, Kawaguchi D (2018) Avifauna of Minami-Iwo-To Island, the Volcano Islands in 2017. Ogasawara Res 44: 217-250. (in Japanese with English summary)

Kawakami K (2019a) The history of anthropogenic disturbance and invasive alien species impact on the indigenous avifauna of the Ogasawara Islands, southern Japan. Jpn J Ornithol 68: 237–262. (in Japanese with English abstract)

Kawakami K (2019b) Minami-Iwo-To: The Last Vestige of Wilderness in the Ogasawara Islands. Glo Envi Res 23: 77-81.

Kawakami K, Suzuki H, Horikoshi S (2021) Avifauna of Kita-iwoto Island, the Volcano Island Group, in 2019. Ogasawara Res 47: 185-200. (in Japanese with English summary)

Kawakami K, Horikoshi K (2022) Recovery or change? Differences between in seabird fauna in island ecosystems before alien mammal disturbance and after alien mammal eradication. Restor Ecol 30(5): e13579.

Kishimoto T (2009) Endangered situation of above-ground animals on the Ogasawara Islands and effect of invasive alien toad, *Bufo marinus*. The nature and insects 44(6): 11-16. (in Japanese)

Momiyama T (1930) On the Birds of Bonin and Iwo-Islands. Bull Biogeogr Soc Jpn, 1: 89-186.

Ono K (1984) Paleontology of seabirds. Marine sciences monthly 16(4): 235-240. (in Japanese)

Sasaki T, Yamada T, Mukai A et al (2018) Altitudinal distribution of large terrestrial isopod and decapod crustaceans on Minami-Iwo-To Island. Ogasawara Res 44: 305-314. (in Japanese with English summary)

Sasaki T, Ameda Y, Horikoshi S, Goto M (2021) Land crabs of Kita-iwoto Island. Ogasawara Res 47: 147-167. (in Japanese with English summary)

Seebohm H (1890) The birds of the Japanese Empire. RH Porter, London.

Shinobe S, Uchida S, Mori H et al (2017) Declining soil Crustacea in a World Heritage Site caused by land nemertean. Sci rep 7(1): 12400.

Shumiya T, Kato H, Takayama K (2018) Forest pattern and changes during the last decade along altitudinal gradients on Minami-Iwo-To Island. Ogasawara Res 44: 91–123.

Sugiura S (2016) Impacts of introduced species on the biota of an oceanic archipelago: the relative importance of competitive and trophic interactions. Ecol Res 31(2): 155-164.

Suzuki K, Suzuki R (2009) Current status of the natural environment of the Ogasawara Islands for World Heritage Registration. Annu Rep Ogasawara Stu 32: 27-47. (in Japanese)
